# Pluronic Micelle-Mediated Tissue Factor Silencing Enhances Hemocompatibility, Stemness, Differentiation Potential, and Paracrine Signaling of Mesenchymal Stem Cells

**DOI:** 10.1021/acs.biomac.1c00070

**Published:** 2021-04-05

**Authors:** Vignesh K. Rangasami, Ganesh Nawale, Kenta Asawa, Sandeep Kadekar, Sumanta Samanta, Bo Nilsson, Kristina N. Ekdahl, Susanna Miettinen, Jöns Hilborn, Yuji Teramura, Oommen P. Varghese, Oommen P. Oommen

**Affiliations:** † Bioengineering and Nanomedicine Group, Faculty of Medicine and Health Technologies, Tampere University, Tampere 33720, Finland; ‡ Translational Chemical Biology Laboratory, Department of Chemistry, Ångström Laboratory, Uppsala University, Uppsala 751 21, Sweden; § Department of Bioengineering, The University of Tokyo, 7-3-1 Hongo, Bunkyo-ku, Tokyo 113-8656, Japan; ∥ Department of Immunology, Genetics, and Pathology, Rudbeck Laboratory, Uppsala University, Uppsala SE-75105, Sweden; ⊥ Department of Chemistry and Biomedical Sciences, Faculty of Health and Life Sciences, Linnaeus University, Kalmar SE-391 82, Sweden; # Adult Stem Cells Group, Faculty of Medicine and Health Technologies, Tampere University, Tampere 33014, Finland; ¶ Research, Development and Innovation Center, Tampere University Hospital, Tampere 33520, Finland; ∇ Polymer Chemistry, Department of Chemistry—Ångström Laboratory, Uppsala University, Uppsala 751 21, Sweden

## Abstract

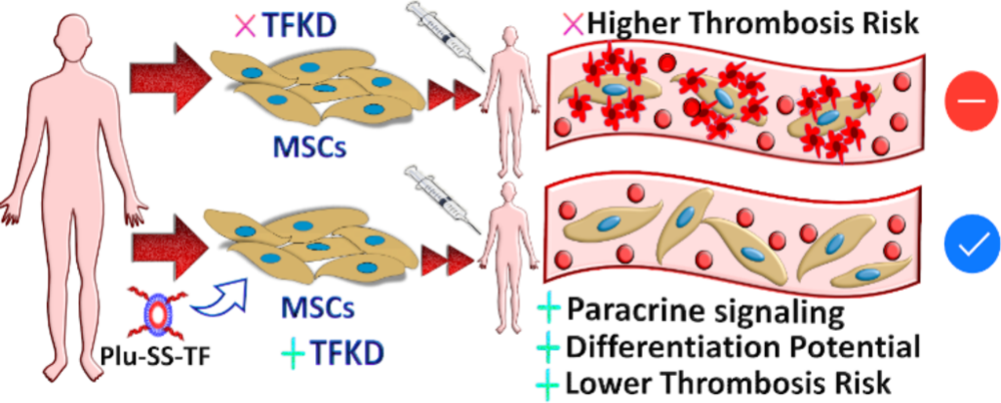

Mesenchymal stem/stromal cells (MSCs) evoke great excitement for treating different human diseases due to their ability to home inflamed tissues, suppress inflammation, and promote tissue regeneration. Despite great promises, clinical trial results are disappointing as allotransplantation of MSCs trigger thrombotic activity and are damaged by the complement system, compromising their survival and function. To overcome this, a new strategy is presented by the silencing of tissue factor (TF), a transmembrane protein that mediates procoagulant activity. Novel Pluronic-based micelles are designed with the pendant pyridyl disulfide group, which are used to conjugate TF-targeting siRNA by the thiol-exchange reaction. This nanocarrier design effectively delivered the payload to MSCs resulting in ∼72% TF knockdown (KD) without significant cytotoxicity. Hematological evaluation of MSCs and TF-KD MSCs in an ex vivo human whole blood model revealed a significant reduction in an instant-blood-mediated-inflammatory reaction as evidenced by reduced platelet aggregation (93% of free platelets in the TF-KD group, compared to 22% in untreated bone marrow-derived MSCs) and thrombin–antithrombin complex formation. Effective TF silencing induced higher MSC differentiation in osteogenic and adipogenic media and showed stronger paracrine suppression of proinflammatory cytokines in macrophages and higher stimulation in the presence of endotoxins. Thus, TF silencing can produce functional cells with higher fidelity, efficacy, and functions.

## Introduction

Mesenchymal stem/stromal cells (MSCs) are adult stem cells that orchestrate immunoregulatory functions and have been extensively evaluated in clinical trials for treating various diseases such as graft-versus-host disease, liver cirrhosis, Crohn disease, stroke, myocardial infarction, allograft rejection, and multiple sclerosis.^1^ MSCs are believed to be immunoprivileged and are transplanted to patients across the major histocompatibility complex barriers. However, recent studies suggest that upon transplantation, patients generate antibodies against these cells, leading to immune rejection.^2,3^ MSCs express C3a and C5a receptors that help in the recruitment of these cells to the injury site.^4^ MSCs also express key regulators such as CD46, CD55, and CD59 that provide defense against the autologous complement.^5^ However, despite these factors, stem cells receive limited protection from complement-mediated cell lysis. Several studies reveal that infusion of MSCs in patients leads to pulmonary thromboembolism mediated by activation of the coagulation cascade^6−8^ with some patients suffering fatal consequences.^9^ Among different factors that drive thrombotic response, the tissue factor (TF, also called CD142 or factor III) encoded by the *F3* gene and expressed on the MSC surface is believed to be the most dominating factor and a key determinant of hemocompatibility.^10^ TF expression activates coagulation and leads to elevated levels of the thrombin–antithrombin (TAT) complex.^7,11^

Careful selection of bone marrow-derived MSCs (BMSCs) that are deficient in TF is therefore proposed as a novel strategy to improve the hemocompatibility of the transplanted cells.^12^ MSCs derived from the adipose tissue (ASCs) express higher TF and show elevated procoagulant activity as compared to the BMSCs.^13^ Therefore, systemic administration of ASCs results in a lower in vivo survival rate than BMSCs.^14^ Hence, there is a pressing need to develop new tools to engineer MSCs that could suppress the instant blood-mediated inflammatory reaction (IBMIR), which significantly suppress the efficacy of MSCs after in vivo infusion. There is also a need to enhance the stemness and differentiation potential of MSCs for safer and effective translation of cell-based products. One of the promising strategies to engineer stem cells with enhanced in vivo survival and paracrine functions is by ex vivo manipulation of these cells. Engineered cell-based therapies have gained prominence over the past decade and even gained approval from the Center for Biologics Evaluation and Research in the United States^15^ and the European Medicines Agency in Europe.^16,17^ There have already been studies using engineered stem cells to treat pancreatic cancer using MSCs armed with the TRAIL gene^18^ to deliver growth factors in an ALS model^19^ and CRISPR edited stem cells to produce erythroid protein to treat disorders that require protein replacement therapy.^20^ As TF expressed on the stem cell surface is one of the key drivers of the coagulation cascade, we hypothesized that effective silencing of procoagulative pathways in MSCs before infusion would potentially suppress the damage caused by IBMIR. Such engineered cells could not only increase cell survival but also display higher fidelity, efficacy, and functions upon transplantation.

We have recently reported the first anionic transfection method to deliver siRNA molecules to MSCs using hyaluronic acid-coated nanoparticles, which could efficiently transfect cells under standard culture conditions^21^ as well as under suspension conditions.^22^ However, such strategies require a significantly higher concentration of RNA for effective knockdown (KD) as complexation with two anionic polymers is not very efficient and the use of excess biopolymer also blocks the nanoparticle uptake. We, therefore, envisioned developing a charge-neutral nanoparticle system where siRNA is covalently conjugated to the nanocarrier. To engineer such a system that safely delivers TF-targeting siRNA to MSCs, we utilized Pluronic F108, a block copolymer possessing a poly(ethylene glycol)-*block* or PEG as a hydrophilic arm and poly(propylene glycol) forming the hydrophobic core that self-assembles to form nanoparticles (NPs).

## Experimental Section

### Synthesis of Pluronic-siRNA Conjugates with Disulfide Linkage

Disulfide functional siRNA was synthesized on an automated solid-phase synthesizer using a thiol-modified solid support to the sense strand of siRNA employing the standard synthesis cycle for RNA. Further, both the strands were deprotected and purified by polyacrylamide gel electrophoresis (PAGE), and the equimolar amount was mixed to form a duplex. To the solution of duplex RNA (50 μL, 2.5 nmol, 25 μM), dithiothreitol (DTT, 10 μL, 50 mM) and H_2_O (40 μL) were added. The reaction mixture was incubated at 37 °C for 2 h. Thereafter, 3 M NaCl (150 μL) was added, followed by H_2_O (150 μL), and the mixture was vortexed and centrifuged down. Then, ethanol (100%, 1000 μL) was added, vortexed, and stored at −20 °C for 18 h. The RNA was micro-centrifuged at 13,000 rpm for 20 min at 4 °C. The supernatant was removed. The pellet was washed with absolute ethanol (100 μL) and micro-centrifuged at 13,000 rpm at 4 °C for 10 min, and the supernatant was removed. The pellet was directly dissolved in disulfide-activated Pluronic solution [368 μL, phosphate-buffered saline (PBS), pH 8, 250 nmol] (detailed description in the Supporting Information). The reaction mixture was incubated at room temperature overnight. Then, the reaction mixture was directly used for conjugation analysis and gene KD experiments.

### Particle Size Distribution by Dynamic Light Scattering

The particle size distribution of the nanoparticles was carried out using a laser granulometer (Zetasizer Nano ZS, Malvern, UK) using a disposable polystyrene cuvette. For the Plu-SS-TF/Ca particles, 100 nM equivalents of siRNA in Plu-SS-TF were added to 50 μL of 100 mM CaCl_2_. This mixture was then vortexed and incubated at room temperature for 10 min. An aliquot of 700 μL of deionized water was added to the solution, and the dynamic light scattering (DLS) experiments were performed. The experiments at 25 °C were performed immediately, whereas the solution was incubated at 37 °C for 30 min before the recordings were done at 37 °C. For the Plu-SS-TF particles without the calcium complexation, 100 nM of siRNA equivalent in Plu-SS-TF was added directly to 750 μL of deionized water, and the DLS measurement was recorded. The surface zeta potential for both the formulations was subsequently measured using a Zetasizer Nano ZS at 25 °C using disposable folded capillary DTS1070 cells.

### Cell Culture

The BMSCs were isolated from a bone marrow aspirate sample obtained from a surgical procedure at the Department of Orthopedics and Traumatology, Tampere University Hospital, with the patient’s consent. The study was conducted in accordance with the Ethics Committee of the Pirkanmaa Hospital District, Tampere (R15174). These BMSCs cells were cultured in α-MEM high glucose (Thermo Fisher Scientific, Vantaa, Finland) with 10% fetal bovine serum (FBS) and 1% penicillin–streptomycin (Penstrep). The cells used in this study were between passages 4 and 6. StemPro osteogenesis and adipogenesis differentiation kits from Gibco were used in the differentiation experiments. THP-1 cells were cultured in RPMI with 10% FBS and 1% Penstrep. Phorbol 12-myristate 13-acetate (PMA) (50 ng/mL) was used to differentiate the THP-1 cells to the M0 state. 500 ng/mL lipopolysaccharide (LPS) was used to activate the M0 cells into the M1 phase.

### Transfection of Cells

Cells were transfected with pyridyl disulfide Pluronic F108 conjugated TF 3 (Plu-SS-TF) siRNA with calcium chloride and RNAiMAX (Thermo Fisher Scientific, Vantaa, Finland). Nanoparticles of Pluronic-linked siRNA and calcium chloride (Plu-SS-TF/Ca) were prepared by adding 50 nM of Pluronic-linked TF siRNA to 25 μL of 100 mM CaCl_2_. They were mixed by vortexing, followed by incubation at room temperature for 10 min. At the end of incubation, the Plu-SS-TF/Ca nanoparticles were added to cells in a single well of a 24-well plate. Cells were also transfected with Pluronic-linked TF siRNA by RNAiMAX (Plu-SS-TF/RNAiMAX). Unconjugated TF siRNA was transfected with both RNAiMAX (TF/RNAiMAX) and Plu-SS and calcium chloride (Plu-SS/TF/Ca) using similar amounts of siRNA and calcium chloride. The cells were incubated for 24 h after transfection at 37 °C and 5% CO_2_. RNA was then isolated and quantitative real-time polymerase chain reaction (qRT-PCR) was performed, as described in the Supporting Information.

### Hematological Studies

Fresh human whole blood was obtained from three healthy volunteers who had not received any medication for at least 10 days before donation, and no heparin was added into the blood. Loops of the polyurethane tubing (an inner diameter of 6.3 mm) with the 2-methacryloyloxyethyl phosphorylcholine (MPC) polymer [poly(MPC-*co*-*n*-butyl methacrylate)] with a 0.30 MPC unit mole fraction were used for the whole blood experiments.^23^ Stainless-steel connectors were coated with the Corline heparin surface (Corline Systems AB, Uppsala, Sweden) according to the manufacturer’s protocol. Loops were composed of the tubing that was closed with surface-heparinized connectors (length: 30 cm, blood volume: 2.5 mL) and were loaded with samples. There were two groups of 1.5 × 10^4^ cells each, the cells after the KD of TF (Plu-SS-TF/Ca) and cells without TF-KD (control MSCs). They were then rotated on a wheel at 50 rpm in a 37 °C cabinet for 1 h. As a negative control, the same volume of the cell culture medium was used. The blood was collected and mixed with ethylenediaminetetraacetic acid (EDTA) (10 mM) and then centrifuged (3400 rpm, 20 min, 4 °C) to collect plasma. The collected plasma was stored at −80 °C before enzyme-linked immunosorbent assay (ELISA) analysis (described in detail in the Supporting Information). These experiments were repeated three times using blood from different donors for each group. Ethical approval was obtained from the regional ethics committee in Uppsala (#2008-264).

### Differentiation Experiments

The cells were transfected with Plu-SS-TF and incubated for 24 h under normal culture conditions. The mediums were then replaced with the osteogenic medium (StemPro Osteogenesis Differentiation Kit from Gibco) and adipogenic mediums separately for 16 days. Osteogenic differentiation of cells was analyzed by alizarin red (Sigma-Aldrich) staining.^24^ Briefly, the cells were fixed with 4% paraformaldehyde for 15 min. They were washed with PBS twice and stained with 2% alizarin red solution for 5 min. The samples were then washed twice with water and observed under a microscope. The adipogenic differentiation of the cells was observed by staining the cells with Nile red (Sigma-Aldrich).^25^ Briefly, the cells were fixed with 4% paraformaldehyde for 15 min. They were washed with PBS twice and stained with 300 nM of Nile red solution. The samples were incubated with the dye for 30 min, after which they were washed with PBS and observed under a fluorescence microscope (Nikon Eclipse Ti2). The expression levels of the osteogenic markers alkaline phosphatase (ALP), osteocalcin (BGLAP), and distal-less homeobox 5 (DLX5) and the adipogenic markers lipoprotein lipase (LPL) and peroxisome proliferator activated receptor gamma (PPARG) were analyzed by qRT-PCR. The expression levels of these Plu-SS-TF/Ca-treated MSCs were compared with untreated controls.

### Condition Medium on THP-1 Cells

The THP-1 cells were first differentiated with 50 ng/mL of PMA (Sigma-Aldrich) for 24 h at 37 °C and 5% CO_2_. The cells were then differentiated 500 ng/mL LPS (Sigma-Aldrich) for 24 h at 37 °C and 5% CO_2_. These cells were then detached with 2 mM EDTA (Sigma-Aldrich) and plated in 24-well plates (60,000 cells/well). These macrophage were incubated with a conditioned medium (CM) collected from the MSCs that were either treated with Plu-SS-TF/Ca or untreated control (CM collected after 2 days). The cells were then incubated for 3 days, after which RNA was extracted, and the expression levels of proinflammatory genes were analyzed by qRT-PCR. The TaqMan primers for IL-1β, TNF, and IL-10 were obtained from Thermo Fisher Scientific, Finland.

### BMSC Stimulation with LPS

Briefly, 50,000 cells were seeded in 24-well plates with 500 μL of α-MEM (Gibco) containing 10% FBS (Gibco, South America) and 1% Penstrep (Gibco) and incubated overnight at 37 °C and 5% CO_2_. The cells were then exposed to 50 nM of the Plu-SS-TF/Ca complex. The cells were then incubated for 48 h at 37 °C and 5% CO_2_, after which LPS (2 μg/mL) was added to the cells. For the purpose of control, one group of cells not treated with Plu-SS-TF/Ca and treated with 2 μg/mL LPS and one group of untreated cells were used. After 24 h of incubation, RNA was extracted, and the expression levels of proinflammatory genes were analyzed by qRT-PCR as mentioned above. The TaqMan primers for IL-1β, TNF, and iNOS were obtained from Thermo Fisher Scientific, Finland.

## Results and Discussion

**Figure 1:**
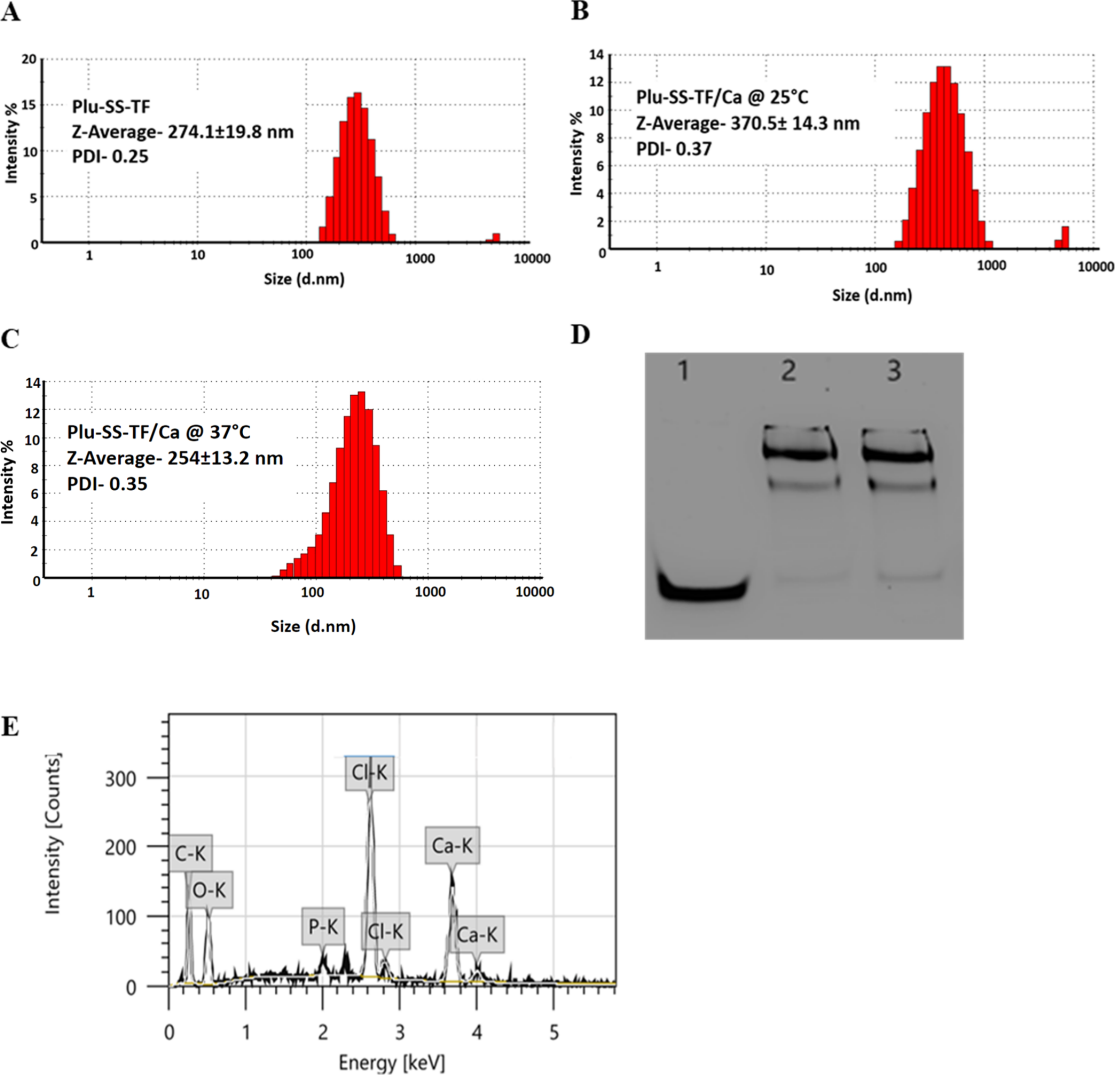
(A) Hydrodynamic size of Plu-SS-TF without calcium complexation and (B, C) with calcium complexation at 25 and 37 °C, respectively. (D) Gel electrophoresis indicating nanoparticle formation. Lane 1 = free siRNA; lane 2 = Plu-SS-TF with calcium complexation; lane 3 = Plu-SS-TF without calcium complexation. (E) EDS elemental mapping indicating the presence of carbon, phosphate, and calcium in Plu-SS-TF/Ca.

**Scheme 1:**
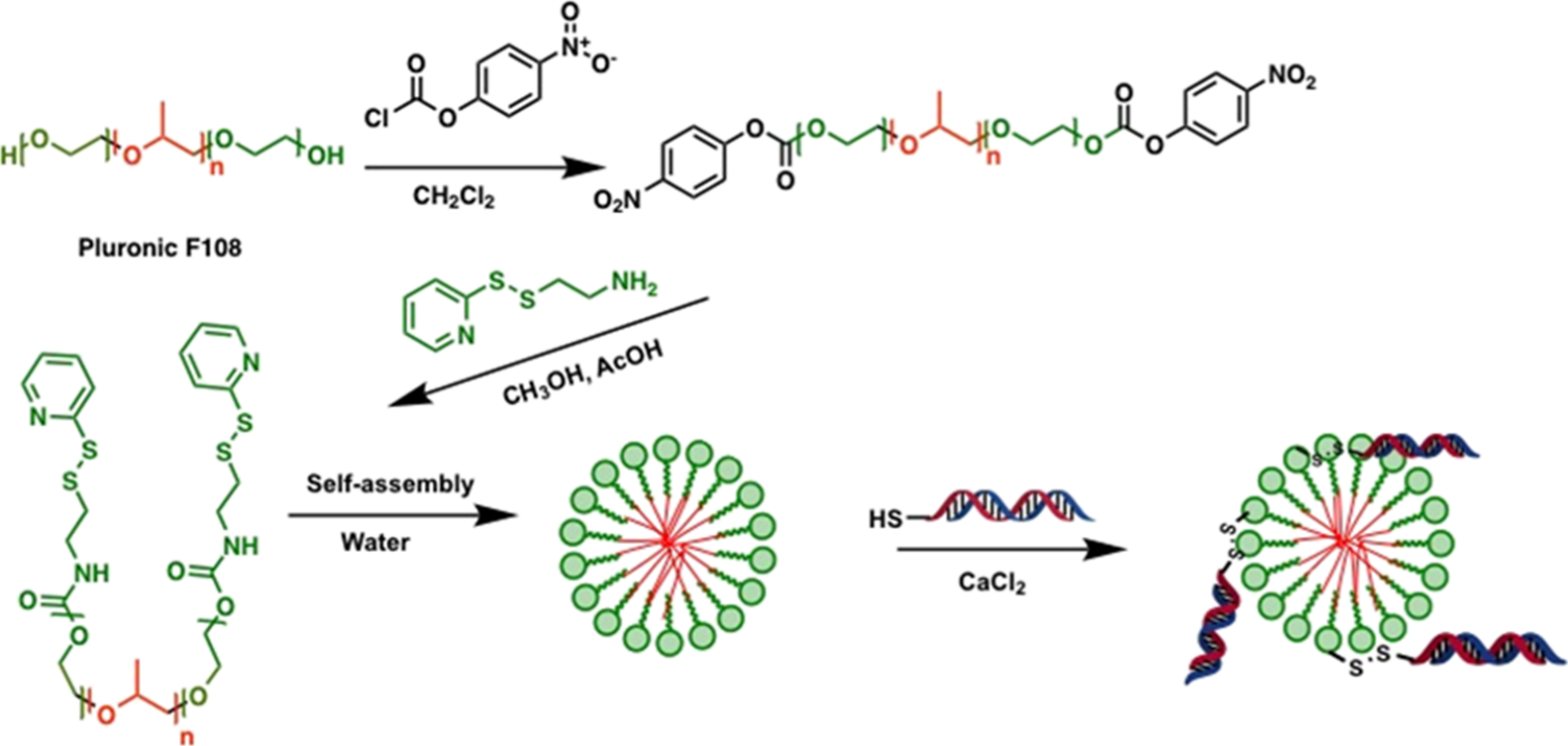
Schematic Description of the Steps Involved in the Synthesis of Plu-SS-TF/Ca

Pluronic-based nanocarriers are effectively used for delivering chemotherapeutic drugs, plasmid DNA, and siRNA molecules as they can coat amphiphilic molecules by hydrophobic interactions.^26−28^ Pluronic F127-coated siRNA/calcium phosphate nanocomplexes are also developed for siRNA delivery to mammalian cells.^29^ Such nanocomplexes are not very efficient as a physical association does not provide good control over siRNA loading efficiency, resulting in inhomogeneous particle distribution. The release of the cargo molecules could also be affected by the temperature (as Pluronic is a thermoresponsive polymer) as well as by the presence of different biomolecules in the milieu. We, therefore, envisaged adopting a covalent grafting strategy where siRNA is covalently conjugated on the hydrophilic arm of the block polymer that would facilitate efficient micelle formation and promote intracellular transport of the cargo molecule. To achieve this aim, we incorporated redox-responsive disulfide groups that provide the dual advantage of fast delivery to the cytosol and glutathione-mediated selective dissociation inside the cells. The excess disulfide groups present on the particle surface after siRNA conjugation can also be exploited for conjugating targeting peptides. To engineer redox-responsive nanocarriers, we first conjugated disulfide pyridyl groups to the terminal hydroxyls present on PEG units by activating the hydroxyls with 4-nitrophenyl chloroformate, followed by nucleophilic displacement reaction with 2-(pyridin-2-yldisulfaneyl)ethan-1-amine, also termed as the amino disulfide pyridyl molecule. We succeeded in obtaining an unprecedented degree of PEG functionalization of over 95% with respect to the available hydroxyls, as verified by UV–vis measurements. In the next step, the disulfide pyridyl-functionalized Pluronic F108 was conjugated with thiol-modified siRNA targeting the TF gene where the thiol groups were incorporated at the 3′ end of the sense strand (Scheme 1). The ratio of siRNA thiol groups and disulfide pyridyl groups was fixed at 10 mol % to achieve quantitative coupling. Indeed, we succeeded in obtaining over 95% conjugation, as evidenced by gel electrophoresis (20% PAGE; Figure 1D). The covalent conjugation resulted in retardation of mobility, indicating higher-molecular-weight species in lane 2 and lane 3, which represents the Pluronic F108-siRNA conjugate (Plu-SS-TF) and the Pluronic F108-siRNA conjugate complexed with 5 mmol Ca^2+^ (Plu-SS-TF/Ca). The native siRNA is presented in lane 1. The siRNA conjugated polymer self-assembled to form micelles that showed a hydrodynamic size of ∼274 nm with unimodal distribution (Figure 1A), which upon complexation with Ca^2+^ displayed a hydrodynamic size of ∼370 nm (Figure 1B), as determined by the DLS experiment. We also confirmed the presence of elemental carbon, phosphate, and calcium in the particles by EDS analysis (Figure 1E). The zeta potential of Plu-SS-TF was estimated to be −12.1 mV, which changed to −0.515 mV upon complexation with Ca^2+^ (Plu-SS-TF/Ca) (Figure S2 in the Supporting Information). This indicates that the addition of Ca^2+^ ions neutralized the net negative charge of phosphates, maintaining an overall near neutral charge. Interestingly, Plu-SS-TF/Ca also displayed thermoresponsive properties as the hydrodynamic size of the micelles reduced from 370 to 254 nm when the DLS experiment was performed at 37 °C instead of 25 °C (Figure 1C). This was also confirmed by scanning electron microscopy (SEM) analysis, which confirmed spherical nanoparticle formation with a ∼200 nm in size (Figure S3 in the Supporting Information).

**Figure 2:**
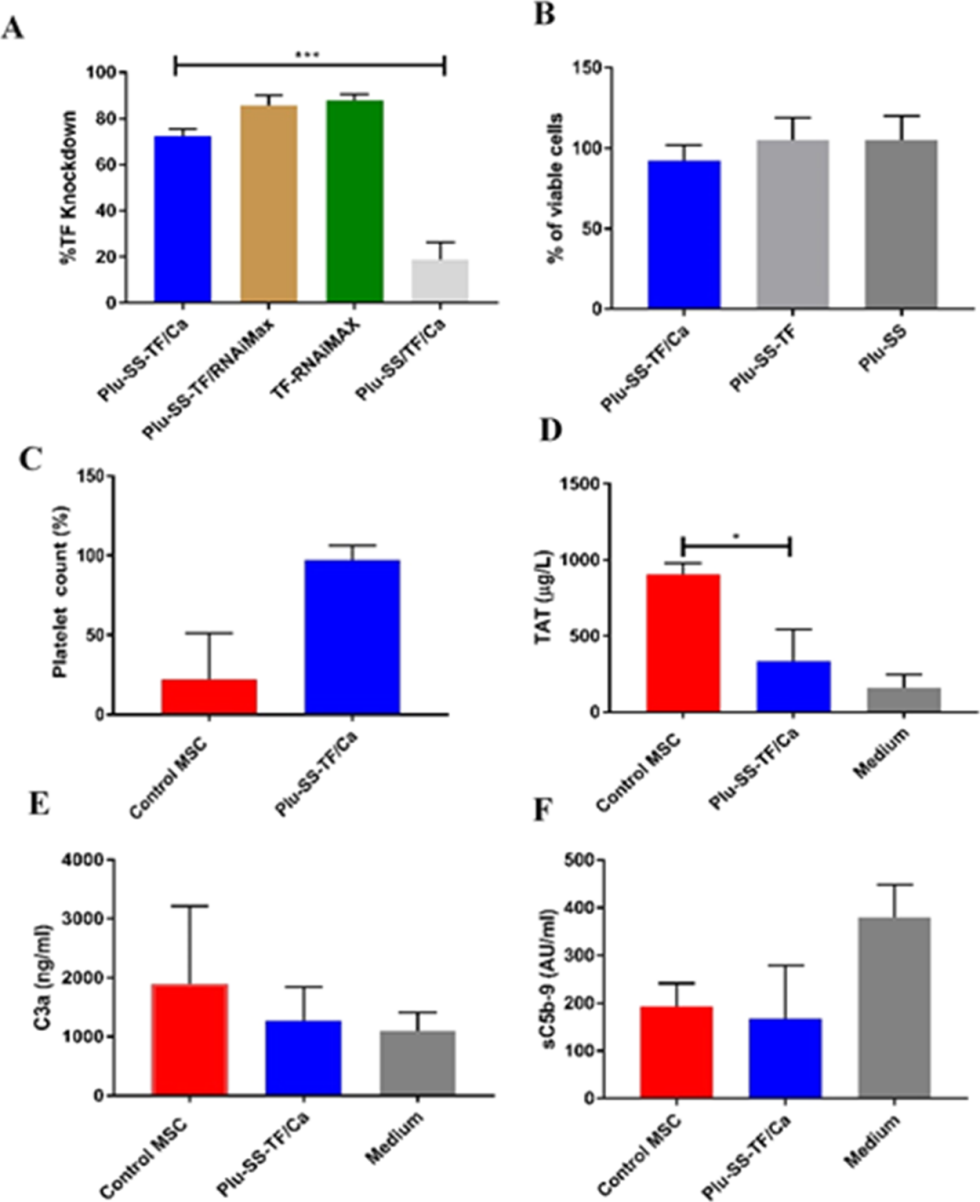
(A) In vitro KD efficiency of different formulations of the nanoparticles with calcium complexation and RNAiMAX. Statistics was done by ANOVA on GraphPad Prism (***P < 0.001). (B) Cell viability percentages obtained for the nanoparticles and their components through the MTT assay. (C) Platelet counts normalized against the growth medium. (D) TAT complex, a marker for coagulation in whole blood (*N* = 3). (E, F) C3a and sC5b-9, markers for complement activation in whole blood (*N* = 3). Statistical analysis was done by the Kruskal–Wallis *T*-test, *P < 0.05.

After successful conjugation of TF siRNA with a releasable disulfide linker to Pluronic micelles (Plu-SS-TF), we performed transfection studies of BMSCs. Since clinical studies with MSCs limit the cell expansion to a maximum of four passages to retain the differentiation potential,^30^ we decided to use BMSCs of higher passages (passages 4–6) in our study. Higher passages are expected to increase the expression of TF,^8^ and we believe that efficient KD of TF in these cells could overcome the deleterious performance of such MSCs, improving its in vivo survival and function. To test the gene silencing efficiency, we first tested the physical coating of the siRNA/Ca^2+^ nanocomplex with the Pluronics F108 polymer functionalized with disulfide pyridyl groups (Plu-SS/TF/Ca) as Pluronics coating of the complex is reported to enhance siRNA delivery.^29^ However, we observed a modest 20% gene silencing with a 50 nM concentration using this system. On the contrary, the positive control experiment with RNAiMAX (TF-RNAiMAX) gave an 86% mRNA silencing efficiency, indicating that the siRNA sequence is effective in silencing the TF gene. Interestingly, the covalently conjugated Plu-SS-TF complexed with Ca^2+^ (Plu-SS-TF/Ca) showed a TF-KD efficiency of 72% when the same concentration of siRNA (50 nM) was used. MSCs are known to be hard-to-transfect cells,^31^ and we believe that the enhanced transfection efficiency of our nanocarrier is due to dithiol groups which are reported to improve the rapid internalization of molecular conjugates.^32^ Earlier reports of high transfection efficiency in MSCs have always come at the cost of poor viability due to the toxicity of the nanocarrier.^31^ This is mainly attributed to the cationic charges on the surface of the particles.^33^ We have previously reported that coating of cationic nanoparticles with anionic polymers not only mitigates cellular toxicity but also assists in the endosomal release of the cargo molecules.^34^ Since the Plu-SS-TF/Ca nanoparticle has a net neutral charge, we anticipated such particles to have minimal toxicity. To evaluate the cytotoxicity of our nanocarrier system, we measured the cellular metabolic activity using the MTT assay, which is an indicator of cell viability, proliferation, and cytotoxicity, and compared it with commercially available RNAiMAX. Interestingly, neither the Pluronic-based micelles (Plu-SS and Plu-SS-TF) nor RNAiMAX showed any toxicity (∼100% cell viability); however, upon complexation with Ca^2+^ (Plu-SS-TF/Ca), the micelles showed ∼90% cell viability (Figure 2B).

Next, we evaluated the effect of TF-KD of BMSCs on the activation of the coagulation cascade using an ex vivo chandler loop model.^35^ We incubated the 1 × 10^4^ BMSCs for 1 h in non-anti-coagulated human whole blood. As anticipated, the TF-KD BMSCs displayed enhanced stability and attenuated platelet aggregation in blood, as evidenced by the availability of ∼93% free platelets (normalized against the growth medium) when compared with untreated BMSCs (22% free platelets) (Figure 2C). This is further corroborated with the TAT complex determination where the TF-KD BMSCs showed significantly lower TAT complex formation when compared to the untreated cells (Figure 2D). Interestingly, we did not see any significant difference in the early and late complement activation markers, namely, C3a and sC5b-9 between the two groups (Figure 2E,F).

**Figure 3:**
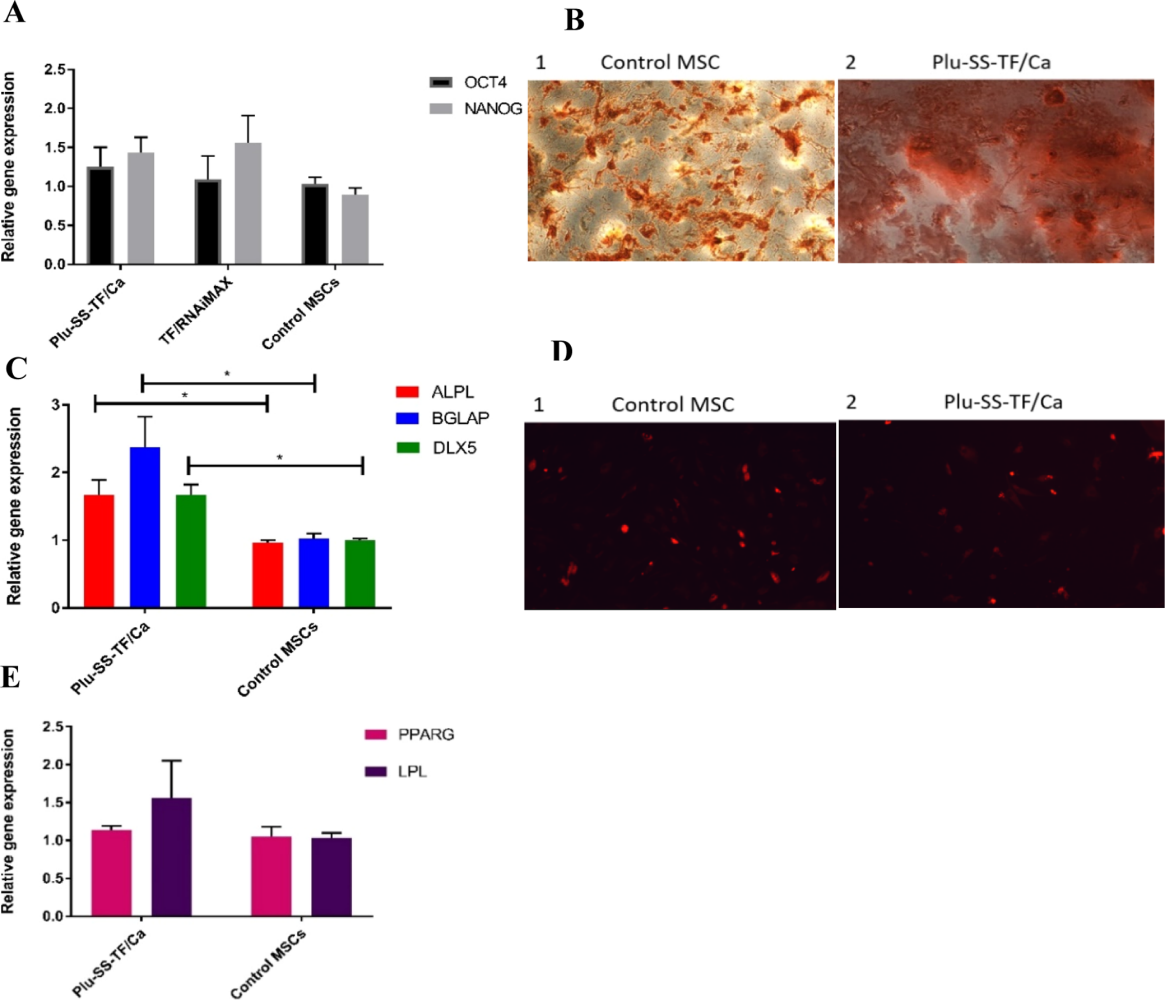
(A) qRT-PCR analysis done to determine the level of OCT4 and NANOG to analyze the stemness of the TF-KD cells and untreated MSCs. (B) qRT-PCR analysis of osteogenic markers. Statistics was done by the Mann–Whitney test on GraphPad Prism (*P < 0.05). (C, D) Differentiation studies of TF-KD MSCs and MSCs after 16 days of culture. (C) Alizarin red staining to detect the presence of calcium deposits of MSCs cultured under osteogenic conditions. (C1) Control MSCs and (C2) MSCs after treatment with Plu-SS-TF/Ca. (D) Nile red staining of MSCs to detect the presence of lipid vacuoles cultured under adipogenic conditions. (D1) Control MSCs and (D2) MSCs after treatment with Plu-SS-TF/Ca. (E) qRT-PCR analysis of adipogenic markers.

As KD of genes in BMSCs could potentially evoke unprecedented responses by altering the paracrine function or by reducing the multipotency of the cells, we investigated the BMSC function by different biochemical methods. We first tested the expression of key cell surface markers that characterize the stem cell physiognomies. It is universally accepted that cells that exhibit positive co-expression of CD105, CD73, and CD90 and are negative for CD45, CD34, and CD14 are characterized as MSCs.^36^ We performed flow cytometry studies to ascertain the impact of TF-KD on the expression of these key cell surface markers. We found that the control BMSCs displayed good population of cells that exhibited high expressions of CD73 (92.1 ± 1.62%), CD105 (91.4 ± 1.8%), and CD90 (82.5 ± 3.2%) and lacked the expression of CD34 (0.4 ± 0.2%) (Figure S4 in the Supporting Information). Gratifyingly, the expression of these markers on the TF-KD BMSC population did not show significant changes. We found that CD73 (89.2 ± 2.2%), CD105 (90.8 ± 1.6%), and CD34 (1.2 ± 0.6%) remained similar, whereas we observed an 8% loss of cells that expressed CD90 (74.1 ± 1.9%) molecules (Figure S3 in the Supporting Information). CD90 is a glycoprotein expressed on the cell surface and is a stem cell marker that signifies the undifferentiated status of MSCs. CD90 (THY-1) controls the differentiation of MSCs as it acts as an impediment toward the pathway of differentiation commitment, and lower expression of CD90 correlates with temporal lineage commitment in vitro.^37^ Thus, lower expression of CD90 as a result of TF-KD could help to increase the differentiation capability of MSCs.^37^ In order to validate the effect of TF-KD on stemness, we measured the expression of OCT4 and the NANOG gene by qRT-PCR as they are the key transcription factors that are crucial for maintaining pluripotency and the self-renewal state.^38^ Interestingly, the qRT-PCR study revealed higher expression of the NANOG gene as a result of TF-KD; however, no significant differences were observed in the OCT4 expression (Figure 3A).

We further validated the impact of TF-KD on the differentiation potential of BMSCs by culturing these cells under osteogenic and adipogenic differentiation conditions. Interestingly, under osteogenic conditions, we observed increased mineralization, as evidenced by higher calcium deposits observed by Alizarin red staining in the TF-KD cells when compared to the control MSCs (Figure 3B). This observation was further validated by qPCR analysis of osteogenic markers under these conditions. We found that the expression of osteogenic markers, namely, ALP, osteocalcein (BGLAP), and DLX5, was on an average 2-fold higher than that in control MSCs under osteogenic conditions (Figure 3C). Interestingly, the TF-KD cells when cultured under adipogenic conditions also showed an increase in the expression of adipogenic markers (LPL and PPARG), albeit not as significantly as under the osteogenic conditions (Figure 3D,E).

**Figure 4:**
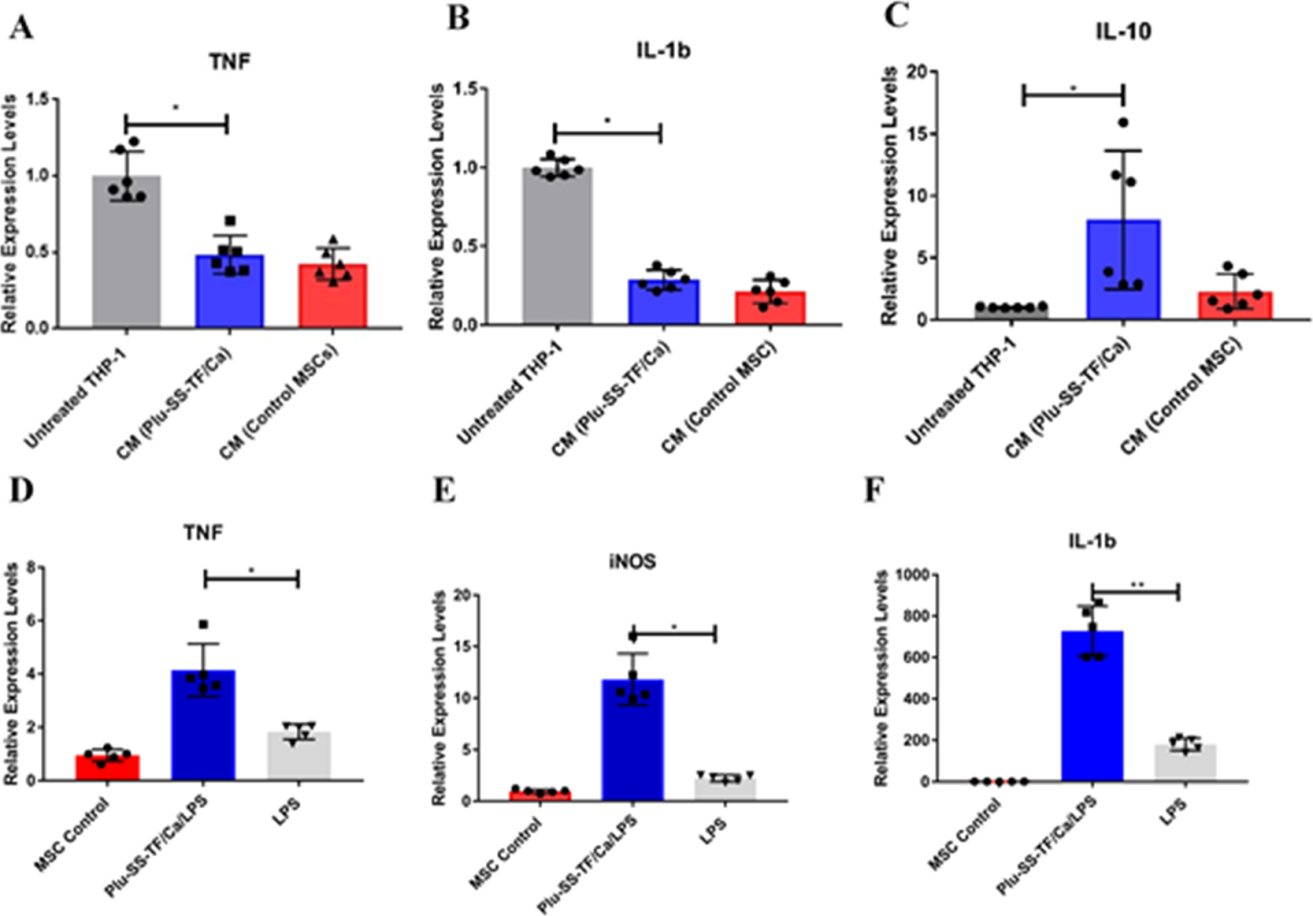
(A–C) Effect of the MSC secretome (CM) on proinflammatory M1 macrophages derived from THP-1 cells. qRT-PCR analysis quantifying the cytokine mRNA levels of (A) TNF, (B) IL-1β, and (C) IL-10. (D–F) Paracrine signaling of MSCs and TF-KD MSCs by direct stimulation with endotoxin LPS (2 μg/mL) relative to the untreated MSC control. qRT-PCR analysis quantifying the cytokine mRNA levels of (D) TNF, (E) iNOS, and (F) IL-1β. Gene expression is relative to β-actin. Statistics was done by the Kruskal–Wallis method (*P ≤ 0.05, **P ≤ 0.01).

We then investigated the immunomodulatory properties of the MSCs by studying the effect of the MSC secretome on proinflammatory M1 macrophages. It is believed that MSCs impart functional benefits in tissue repair and mitigate inflammation by secreting soluble factors by paracrine signaling.^39,40^ In order to assess the immunosuppressive nature of the TF-KD MSCs, we exposed the CM of TF-KD MSCs and control MSCs to the THP-1 human monocyte cell line that was differentiated to the proinflammatory M1 phenotype. We subsequently analyzed the expression of proinflammatory cytokines, namely, TNF and IL-1β as well as the IL-10 cytokine, a master regulator of anti-inflammatory response. As anticipated, incubation of M1 macrophages with the secretome in condition media from TF-KD MSCs and control MSCs suppressed the production of the proinflammatory cytokines TNF and IL-1β, relative to M1 macrophages that were not exposed to the condition media (control) (Figure 4A–C). This suggests that the TF-KD MSCs retained the immunosuppressive ability similar to untreated MSCs. Surprisingly, we observed an increase in the production of the anti-inflammatory IL-10 cytokine when the M1 macrophages were treated with conditioned media from the TF-KD cells. IL-10 is a broad anti-inflammatory cytokine that suppresses the activity of other proinflammatory immune cells and subsequently regulates T-helper cell (Th1 and Th2) responses^41^ and plays an important role in tissue regeneration,^42^ mitigation of liver injury,^43^ and alleviation of fibrosis.^44^ This suggests that the TF-KD MSCs were partially superior to control MSCs in the resolution of inflammation.

Finally, we examined the paracrine capability of BMSCs by directly stimulating the cells with endotoxin LPS (Figure 4D–F). We first silenced TF using Plu-SS-TF/Ca, and after 48 h, we treated the cells with LPS. Surprisingly, TF-KD MSCs displayed significantly higher expression of proinflammatory cytokine TNF, IL-1β, and iNOS than the untreated BMSCs control. This clearly suggests that the TF-KD MSCs were more sensitive to stimulation.

## Conclusions

In conclusion, we have engineered a novel Pluronic-based nanocarrier for the efficient delivery of siRNA to stem cells. We exploited this system to deliver siRNA that targets TF or CD142 in BMSCs and evaluated the procoagulative activities in human whole blood as well as its differentiation and paracrine function. The Pluronic nanoparticle-mediated siRNA delivery displayed over 70% TF silencing without eliciting any significant cytotoxicity. Hematological evaluation of BMSCs and TF-KD MSCs in an ex vivo human whole blood model revealed a significant reduction in IBMIR, as evidenced by reduced platelet aggregation (93% of the free platelets in the TF-KD group as compared to only 22% in untreated BMSCs) and TAT complex formation. Effective silencing of TF enhanced the differentiation of BMSCs in osteogenic and adipogenic media, as evidenced by increased mineralization as well as higher expression of ALP, BGLAP, and DLX5 genes relative to untreated BMSCs. The TF-KD BMSCs displayed higher paracrine signaling as they exhibited enhanced stimulation upon exposure to endotoxin. This is evident from higher expression of TNF, iNOS, and IL-1β. Furthermore, the soluble factors produced by TF-KD BMSCs and untreated BMSCs competently suppressed the proinflammatory cytokines such as TNF and IL-1β and increased the production of the anti-inflammatory IL-10 cytokine when supplemented to proinflammatory M1 macrophages. Collectively, this work provides compelling evidence that efficient silencing of TF in BMSCs by Plu-SS-TF micelles provides a novel strategy to minimize the risk associated with thrombotic complications. Surprisingly, the engineered cells also exhibit enhanced immunosuppressive properties and superior paracrine functions and differentiation potential, which may increase the patient safety and benefits in existing BMSC-based therapies.

## Supporting Information Available

Synthesis protocol for synthesizing Pluronic derivatives; siRNA sequence; zeta potential data; PAGE assay; SEM protocol and data; qRT-PCR; cytotoxicity study; ELISAs for coagulation and complement activation markers; and flow cytometry study for stem cell marker assessment (PDF)
