# Supplementary material for: Pluronic Micelle-Mediated Tissue Factor Silencing Enhances Hemocompatibility, Stemness, Differentiation Potential, and Paracrine Signaling of Mesenchymal Stem Cells
Source: Biomacromolecules. 2021 Apr 5;22(5):1980–9. doi: 10.1021/acs.biomac.1c00070 (PMC8154246; doi:10.1021/acs.biomac.1c00070)
Supplement: Supplementary file 1 — bm1c00070_si_001.pdf [file bm1c00070_si_001.pdf]

Supporting Information

Pluronic micelles mediated tissue factor silencing enhances hemocompatibility, stemness, differentiation potential, and paracrine signaling of mesenchymal stem cells

*Vignesh K. Rangasami,[a] Ganesh Nawale,[b] Kenta Asawa,[c] Sandeep Kadekar,[b] Sumanta Samanta,[a] Bo Nilsson,[d] Kristina N Ekdah [d,e] Susanna Miettinen,[f,g] Jöns Hilborn,[c] Yuji Teramura,[c,d] Oommen P. Varghese,[c] and Oommen P. Oommen[a,]**

[a] Bioengineering and Nanomedicine group, Faculty of Medicine and Health Technologies, Tampere University, 33720, Tampere, Finland. *Corresponding author- Email- oommen.oommen@tuni.fi.

[b] Translational Chemical Biology Laboratory, Department of Chemistry, Ångström Laboratory, Uppsala University, 751 21, Uppsala, Sweden.

[c] Department of Bioengineering, The University of Tokyo, 7-3-1 Hongo, Bunkyo-ku, Tokyo, 113-8656, Japan.

[d] Department of Immunology, Genetics and Pathology, Rudbeck Laboratory, Uppsala University, SE-75105, Sweden.

[e] Department of chemistry and biomedical sciences, Faculty of health and life sciences, Linnaeus University, Kalmar, Sweden.

[f] Adult Stem Cells Group, Faculty of Medicine and Health Technologies, 33014, Tampere University, Finland.

[g] Research, Development and Innovation center, Tampere University Hospital, 33520, Tampere, Finland

[h] Polymer Chemistry, Department of Chemistry - Ångström Laboratory, Uppsala University, 751 21, Uppsala, Sweden.

Keywords: Mesenchymal stem cells; pluronic micelles; hemocompatibility; immunology; siRNA delivery, Cell therapy.

Materials and methods

Preparation of pyridyl disulfide ligand (2)

Mercaptoethylamine (cysteamine) hydrochloride 1 (2.288 g, 20.1408 mmol) was dissolved in methanol (17.5 ml) followed by addition of glacial acetic acid (1.6 ml). Above solution was then added dropwise to a stirred solution of 2,2- dithiopyridine (8.815 g, 40.2817 mmol) in methanol (42 ml). Reaction mixture (RM) was stirred for 48 h at room temperature (RT) and product was precipitated by the addition of diethyl ether (200 ml). The precipitated product was further dissolved in a small volume of methanol and was again reprecipitated by diethyl ether followed by purification with silica column chromatography (10 % MeOH/ EtOAc) to afford white solid compound 2 (3.300 gm 73 %). 1H NMR (400 MHz, D2O) δ ppm 8.31 - 8.50 (m, 1 H) 7.78 - 7.84 (m, 1 H) 7.70 - 7.75 (m, 1 H) 7.28 - 7.33 (m, 1 H) 3.30 - 3.35 (m, 3 H) 3.06 - 3.11 (m, 2 H).

Preparation of activated pluronic with 4-nitrophenyl chloroformate (4)

Pluronic F108 **3** (2 g, 0.14 mmol) was dissolved in 10 ml of dichloromethane (DCM), and to this solution slowly added 4-nitrophenyl chloroformate (170 mg, 0.84 mmol. The reaction mixture was stirred at room temperature for overnight and product was precipitated with diethyl ether. Precipitation was repeated for three times to afford activated pluronic **4** (2.025 gm 99 %). The degree of activation was determined using UV-VIS spectrophotometer (402 nm, molar extinction coefficient of 18400 cm^{-1} M^{-1}). Briefly, the activated pluronic **4** was dissolved in 0.1 M NaOH (1 mg/1 ml). After 1 h, its absorbance was measured using UV-Vis at 402 nm which showed that the 4-nitrophenyl modification of PEG was quantitative.

Synthesis of Pluronic F108 pyridyl disulfide derivative (5)

Ligand **2** (154 mg, 0.69 mmol) dissolved in water was treated with saturated $NaHCO_3$ to remove the counter ion. It was subsequently extracted with DCM and then dried over Na_2SO_4 followed by vacuum concentration to get the yellowish oily compound. The extracted ligand was subsequently dissolved in 1 mL of dry DCM, and was added to activated pluronic **4** (1 g, 0.069 mmol) that was dissolved in 10 mL of dry DCM. The reaction mixture was refluxed for overnight. The reaction mixture was evaporated using a rotary evaporator and then diluted with methanol: water (1:1, v/v, 10 ml) and dialyzed (3500 Da cutoff) against 2 L of deionized water for two days. Product **5** was finally recovered by lyophilization to get white solid (0.958 g, 92 %). The degree of modification was determined using UV (343 nm, molar extinction coefficient of 8060 cm^{-1} M^{-1}). The UV absorbance of product **5** (1 mg/mL PBS buffer, pH 9) was measured before as well as 10 min. after the addition of 0.1 mL of DTT (15 mg/mL PBS, pH 9). The degree of modification was quantitative.

A

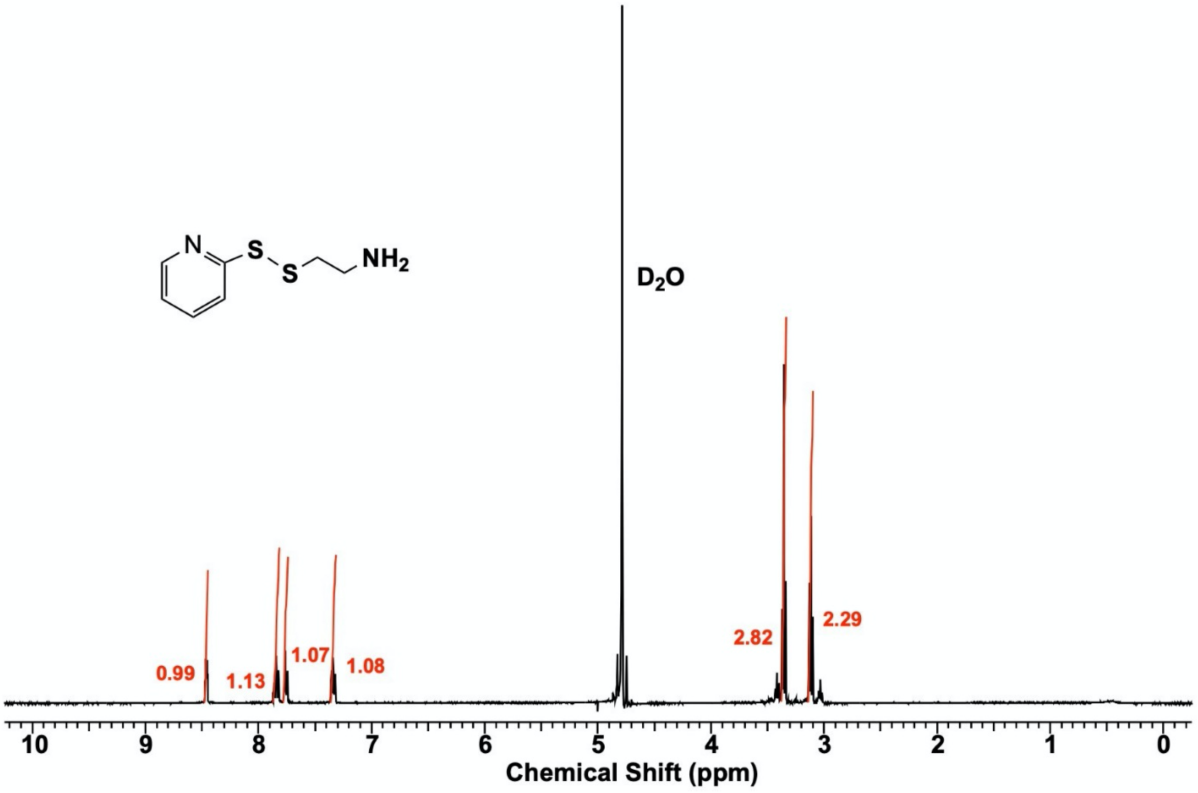

B

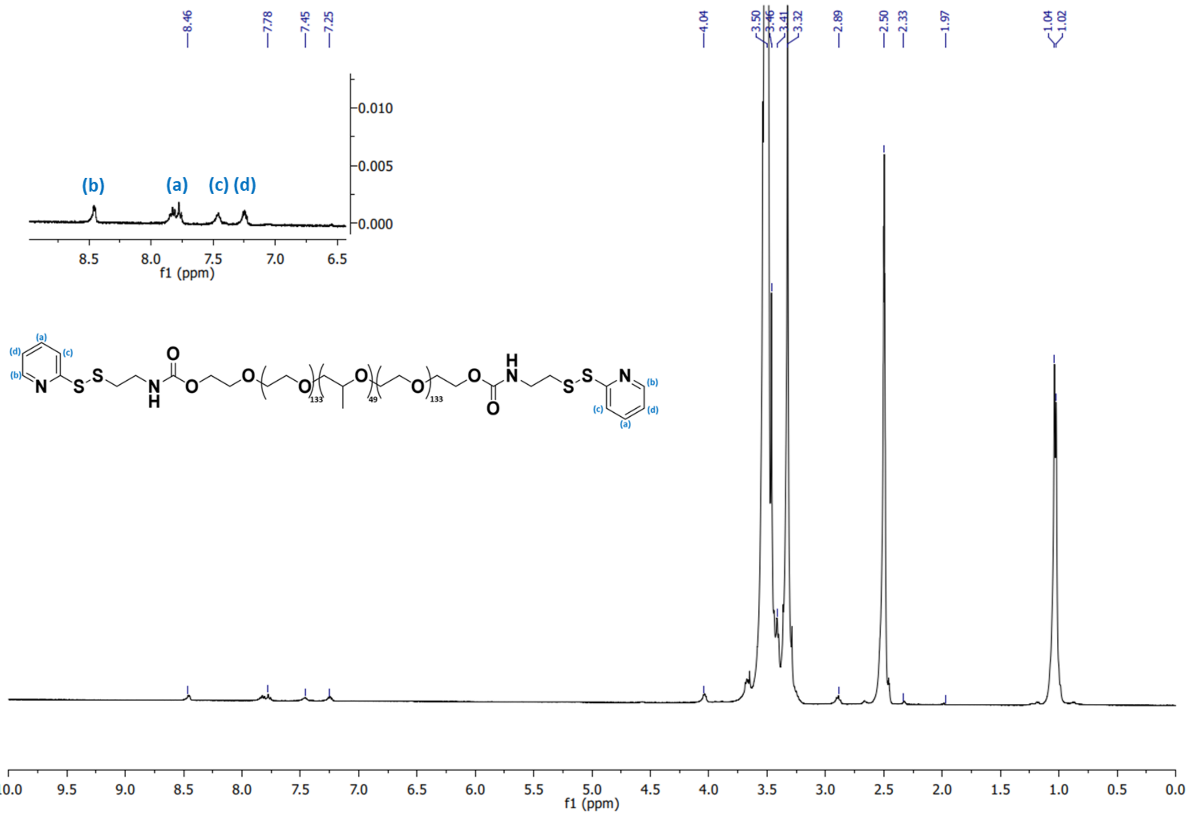

C

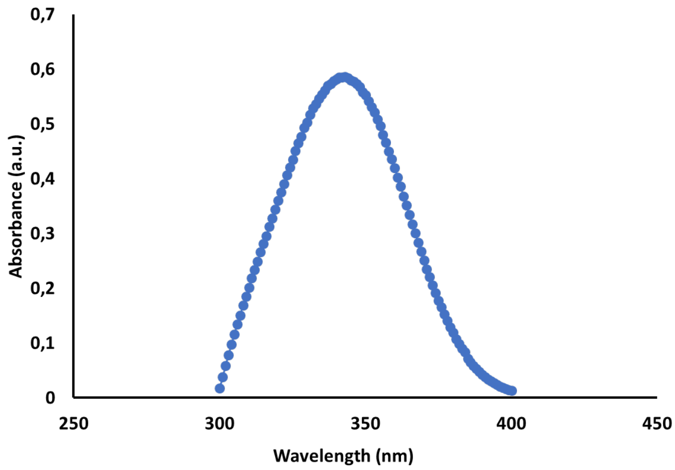

Figure S1: A) The ^{1}H NMR spectra for pyridyl disulfide ligand **2**. B) ^{1}H NMR spectra for Pluronic F108 pyridyl disulfide derivative **5**. C) UV-Vis spectra for Pluronic F108 pyridyl disulfide derivative **5**

Table S1: Sequence of the tissue factor siRNA used for the studies.

TF siRNA Antisense strand	5′-AAGUGUAUAAAUUAAGUCC[dT][dT]-3′
TF siRNA Sense Strand with thiol modification	5′-GGACUUAAUUUAUACACUU[dT][dT]-S-S- 3′

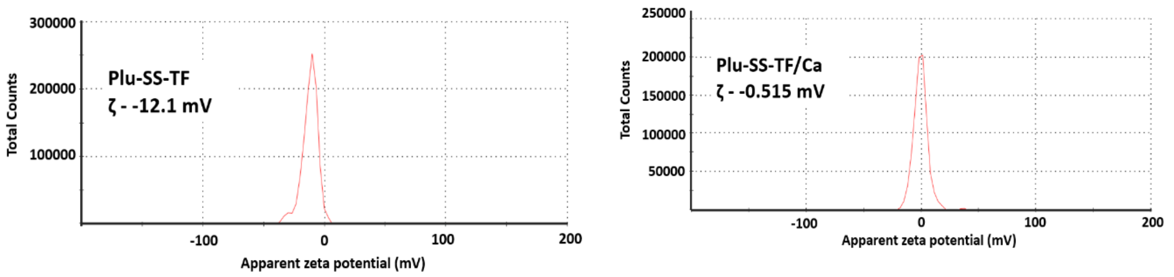

Figure S2: Zeta potential of Plu-SS-TF and Plu-SS-TF/Ca at 25°C in water.

Polyacrylamide gel electrophoresis assay

For the gel electrophoresis, a native 20% polyacrylamide gel was prepared, and Plu-SS-TF/Ca, Plu-SS-TF and free TF siRNA were loaded. The concentrations of samples were kept such that the siRNA concentration was 100nM. Prior to loading the particle complex onto the gel, an equal amount of 2 X RNA loading buffer (Cat# R0641, Thermo Fisher Scientific) was added and the mixture were loaded on to respective wells. The analysis was carried out using 2 W power supply for 2 h and 1 × TBE (89 mM of each Tris and boric acid and 2 mM of EDTA, pH 8.3) was used as running buffer. Thereafter, gels were stained with SYBR™ Gold Nucleic Acid Gel Stain (Cat # S11494, Thermo Fisher Scientific) and visualized under UV.

Scanning Electron Microscopy

The morphology and elemental composition of the Plu-SS-TF/Ca nanoparticles was determined using an SEM (scanning electron microscope) (Jeol IT500) and EDS operating at 15 kV. Prior to the analysis, the samples were diluted in deionised water and drop casted on to

the carbon tabs (Agar Scientific, Stanstead, UK). The samples were then allowed to dry at room temperature in a desiccator overnight. As the samples were non-conductive, a thin layer of conductive carbon (~5 nm) was evaporated on to the samples to make them suitable for SEM analysis.

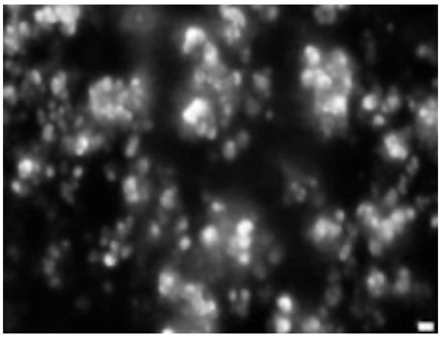

Figure S3: SEM analysis showing the presence of particles after the calcium complexation. (Scale bar- 200 nm)

Quantitative Real-time-PCR

24 hours post transfection the cells were lysed, and RNA was extracted using RNeasy Plus Mini kit from Qiagen. 100 nanogram of the total RNA was used to make the cDNA. The cDNA was prepared using Maxima First Strand cDNA kit according to manufacturer's protocol (Thermo Fisher Scientific, Vantaa, Finland) and qRT-PCR was performed with cDNA and TaqMan® Fast Advanced Master Mix (2X) (Applied Biosystems). The real-time PCR reactions were carried out with 10 μL of 2X TaqMan® Universal PCR Master Mix, no AmpErase® UNG (Applied Biosystems, USA), 2 μL cDNA, and 1 μL of TaqMan gene-specific assay mix (TF and β-actin)(Applied Biosystems) in a 20 μL final reaction volume. Reference gene, β-actin (ACTB) (Taqman primers, Thermo Fisher, Finland) were selected as a control for normalization of real-time PCR data. The amplification was carried out using the Biorad CFX1000 (Bio-rad) using a 40-cycle program. The CFX manager software automatically calculates the raw Ct (cycle threshold) values. Data from samples with a Ct value equal to or below 33 were further analyzed. Samples were normalized relative to endogenous

control and differences in cycle number thresholds were calculated using comparative quantitation 2−ΔΔCT method (also called the ΔΔCT method), which is commonly used for analyzing siRNA induced gene knockdown efficiency.

Cytotoxicity studies

Briefly, 5000 cells were seeded in 96-well plates with 100μL α-MEM (Gibco) containing 10% fetal bovine serum (Gibco, South American) and 1% penicillin-streptomycin (Gibco) and incubated overnight at 37 °C and 5% CO_2. The cells were then exposed to the Plu-SS-TF/Ca complex and Plu-SS-TF without the calcium. The concentration of the Plu-SS-TF depended on the siRNA concentration (50 nM). Equivalent amount of Plu-SS was taken from a stock of 3.2 mg/mL and added to the cells. The cells were then incubated for 48 h at 37 °C and 5% CO_2, after which MTT (5 mg/mL) was added to the cells. After 4 h of incubation 150 μL of DMSO was added to each of the wells and the plates were shaken well. The absorbance was measured using a microplate reader. Results were expressed as percent viability = [A540 (treated cells)-background/A540 (untreated cells)-background] × 100.

Enzyme-linked immunosorbent assays (ELISAs) for coagulation and complement activation markers

PBS containing 1% (w/v) bovine serum albumin (BSA) and 0.05% (v/v) Tween 20 was used as the dilution buffer, PBS containing 0.05% Tween 20 as the washing buffer, and TMB+ substrate chromogen (Dako, Glostrup, Denmark) as the color substrate. Plasma levels of thrombin-antithrombin (TAT) and complement 3a (C3a) and sC5b-9 were analyzed by sandwich ELISA. TAT was analyzed using a commercially available kit from Enzyme Research Laboratories (South Bend, IN, USA). TAT was captured in wells coated with anti-human thrombin antibody diluted 1/20. Horseradish peroxidase (HRP)-conjugated anti-human

antithrombin (AT) antibody diluted 1/20 was used for detection. Pooled human serum diluted in normal citrate-phosphate-dextrose plasma was used as a standard. C3a levels were determined and captured using mAb 4SD17.3, biotinylated polyclonal anti-C3a, and HRP-conjugated streptavidin (GE Healthcare) for detection. sC5b-9 was determined using the anti-neoC9 mAb aE11 (Diatec Monoclonals AS, Oslo, Norway) for capture, and polyclonal anti-C5 antibody (Acris, Herford, Germany) and HRP-conjugated anti-rabbit IgG (Dako) for detection. The assay was calibrated against a commercially available kit (MicroVue, Quidel Corp, Santa Clara, CA, USA).

Flow cytometry for stem cell marker assessment

Cells (50000) in number were plated in 24 well plates and were cultured in normal culture conditions. Plu-SS-TF was added to the cells 18-24 h after plating. The cells were incubated for 24 h after which they were trypsinized and collected for flow cytometry. The cells were incubated with antibodies against a panel of MSC positive and negative markers that included CD34, CD73, CD90 and CD105. The samples were then washed, centrifuged and re-suspended in dPBS+10% FBS solution and analyzed by flow cytometry. CD34-APC was from Immunotools, CD73-PE and CD90-APC were from BD Pharmingen and CD105-PE was obtained from R&D systems. Flow cytometry was performed in a BD Accuri C6 instrument.

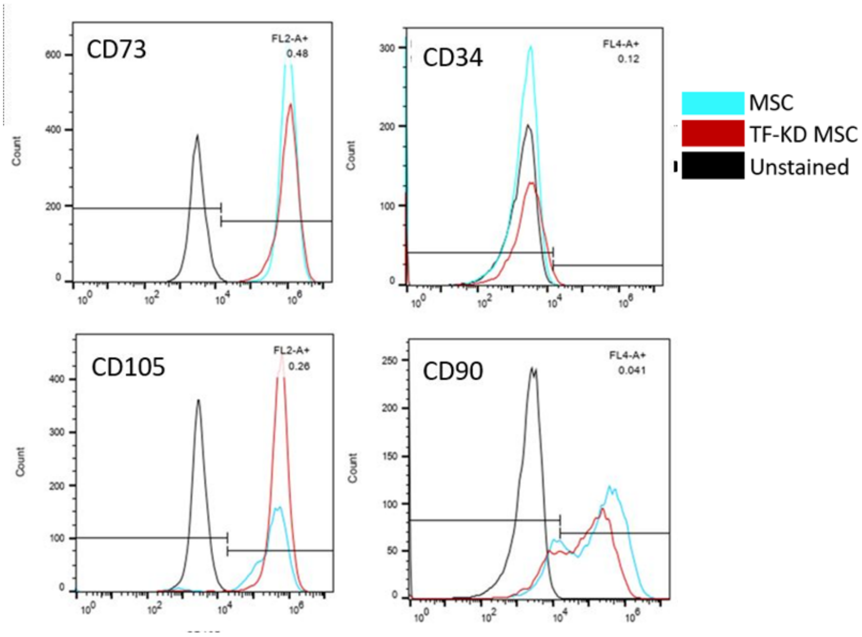

Figure S4: Flow cytometry histogram for key BMSC markers namely; CD73, CD34, CD105 and CD90

Expression of stemness markers

Briefly, 50000 cells were seeded in 24-well plates with 500μL α-MEM (Gibco) containing 10% fetal bovine serum (Gibco, South American) and 1% penicillin-streptomycin (Gibco) and incubated overnight at 37 °C and 5% CO_2. The cells were then exposed to the 50 nM of Plu-SS-TF/Ca complex and Plu-SS-TF/RNAiMAX. The RNA was extracted 24 hours later using RNeasy Mini plus kit from Qiagen, Finland. The cDNA was prepared and qRT-PCR performed as mentioned above. The TaqMan primers for OCT4 and NANOG were obtained from Thermo Fisher Scientific, Finland.
